# Renal angiotensin I-converting enzyme-deficient mice are protected against aristolochic acid nephropathy

**DOI:** 10.1007/s00424-022-02779-4

**Published:** 2022-12-15

**Authors:** Annett Juretzko, Antje Steinbach, Jeannine Witte, Anke Hannemann, Bärbel Miehe, Florian Siegerist, Carmen Wolke, Sylvia Stracke, Rainer Rettig

**Affiliations:** 1 Institute of Physiology, University Medicine, Greifswald, Germany; 2 Institute of Clinical Chemistry and Laboratory Medicine and DZHK (German Centre for Cardiovascular Research), Partner Site Greifswald, University Medicine, Greifswald, Germany; 3 Institute of Anatomy and Cell Biology, University Medicine, Greifswald, Germany; 4 Institute of Medical Biochemistry and Molecular Biology, University Medicine, Greifswald, Germany; 5 Clinic for Internal Medicine A, University Medicine, Greifswald, Germany

**Keywords:** Aristolochic acid I, Angiotensin I-converting enzyme 2, Angiotensin (1–7), Chronic kidney disease, Renin-angiotensin system

## Abstract

The renal renin-angiotensin system (RAS) is involved in the development of chronic kidney disease. Here, we investigated whether mice with reduced renal angiotensin I-converting enzyme (ACE^−/−^) are protected against aristolochic acid nephropathy (AAN). To further elucidate potential molecular mechanisms, we assessed the renal abundances of several major RAS components. AAN was induced using aristolochic acid I (AAI). Glomerular filtration rate (GFR) was determined using inulin clearance and renal protein abundances of renin, angiotensinogen, angiotensin I-converting enzyme (ACE) 2, and Mas receptor (Mas) were determined in ACE^−/−^ and C57BL/6J control mice by Western blot analyses. Renal ACE activity was determined using a colorimetric assay and renal angiotensin (Ang) (1–7) concentration was determined by ELISA. GFR was similar in vehicle-treated mice of both strains. AAI decreased GFR in controls but not in ACE^−/−^ mice. Furthermore, AAI decreased renal ACE activity in controls but not in ACE^−/−^ mice. Vehicle-treated ACE^−/−^ mice had significantly higher renal ACE2 and Mas protein abundances than controls. AAI decreased renal ACE2 protein abundance in both strains. Furthermore, AAI increased renal Mas protein abundance, although the latter effect did not reach statistical significance in the ACE^−/−^ mice. Renal Ang(1–7) concentration was similar in vehicle-treated mice of both strains. AAI increased renal Ang(1–7) concentration in the ACE^−/−^ mice but not in the controls. Mice with reduced renal ACE are protected against AAN. Our data suggest that in the face of renal ACE deficiency, AAI may activate the ACE2/Ang(1–7)/Mas axis, which in turn may deploy its reno-protective effects.

## Introduction

In the second half of the twentieth century, regional outbreaks of progressive interstitial nephritis were reported in several countries worldwide including China, the Balkan states, and Belgium; the etiology of which could later be identified as being associated with the ingestion of aristolochic acid (AA) [17, 34]. The compound is contained in several plants or plant products. The AA-induced renal disease was initially named according to the source of the compound as Chinese herb nephropathy (CHN) or to the region of disease outbreak as Balkan-endemic nephropathy (BEN) and is now generally referred to as aristolochic acid nephropathy (AAN). Although AAN can be prevented by avoiding products containing AA, intoxications with this compound are still frequently reported worldwide [17, 34, 70, 74].

Plant-derived AA is a mixture of two similar components, AAI and AAII. Chemically, AAI differs from AAII by the presence of an O-methyl group in position 8 of the molecular tetracyclic structure. In an experimental study on mice [60], only AAI, but not AAII, was capable of inducing nephrotoxicity. Upon oral ingestion, AAI is readily absorbed from the gastrointestinal tract. In blood plasma, albumin-bound AAI escapes glomerular filtration [21, 60] and reaches proximal tubular epithelial cells (PTEC) on their abluminal side. The compound is then taken up into the cytoplasm via organic anion transporters (OATs), where it may accumulate. Intracellular AAI has been shown to form DNA adducts and to activate various signal transduction pathways [8, 28, 37–39] that may ultimately lead to cellular damage and cell death. Although there has been considerable progress in recent years toward a better understanding of these pathways [2, 46, 53, 59, 69], the molecular mechanisms mediating the cytotoxic effects of AAI are currently not well understood.

There is ample clinical [19, 30, 40, 43, 50, 54] and experimental [41, 45, 55] evidence that the renal renin-angiotensin system (RAS) plays a major role in several forms of renal insufficiency. Thus, the renal RAS mediates pro-inflammatory, pro-fibrotic, and pro-atherosclerotic effects that may not only promote renal disease but may have pathophysiological consequences beyond the kidney to affect systemic conditions such as arterial hypertension. In this regard, it has recently been shown that mice with renal angiotensin I-converting enzyme (ACE) deficiency but normal plasma ACE activity were protected against angiotensin II-induced [26] as well as N(ω)-nitro-L-arginine methyl ester (L-NAME)-induced hypertension [25].

According to the classical paradigm, angiotensin (Ang) II, acting mostly via the angiotensin II type 1 receptor (AT_1_R), is the only effector peptide of the RAS. More recently, a second effector peptide, Ang(1–7), acting via the Mas receptor (Mas), has been described that appears to counteract many of the classical AngII effects, including those mentioned above, thus conferring essentially beneficial effects on the kidney and other organs [57]. The heptapeptide Ang(1–7) is derived from the octapeptide AngII by the action of a monocarboxypeptidase named ACE2. Alternatively, ACE2 may convert the decapeptide AngI [Ang(1–10)] to the nonapeptide Ang(1–9), which in turn is converted to Ang(1–7) by classical ACE.

Given the well-known detrimental effects of the classical ACE/Ang II/AT_1_R axis on the kidney and the emerging reno-protective effects of the alternative ACE2/Ang(1–7)/Mas axis, the present study was designed to investigate whether renal ACE deficiency may convey a protective effect against experimentally induced renal insufficiency. To address this question, we used the C57BL/6 J-*tm*^*(ACE3/3)*^ mouse strain (ACE^−/−^ mice), which is a genetically engineered strain with severe ACE deficiency in the kidney and most other organs. As ACE gene transcription was set under the control of the albumin promoter, these mice show high ACE expression in hepatic tissue and virtually normal serum ACE activity [9, 26]. Of note, ACE^−/−^ mice have been reported to have normal blood pressure and normal urinary concentrating ability [9].

As an experimental paradigm, we opted for AAN, since this form of renal insufficiency has only emerged quite recently and its pathophysiological mechanisms are not yet sufficiently understood. Furthermore, to elucidate potential molecular mechanisms, we assessed the renal abundances of major components of the two alternative RAS axes.

## Materials and methods

### Experimental animals

The renal ACE-deficient C57BL/6J-*tm*^*(ACE3/3)*^ mouse strain (ACE^−/−^ mice) was kindly provided by Dr. R. A. Gonzalez-Villalobos (Los Angeles, CA, USA). The generation of ACE^−/−^ mice has been described elsewhere [9]. Briefly, ACE gene expression was set under the control of the albumin promoter, resulting in ACE gene expression being restricted mainly to the liver. Nevertheless, there was some minor renal ACE expression in the ACE^−/−^ mice (about 14% of that in wildtype controls) [47], which may be due to some residual activity of the albumin promoter cassette in the kidney. The ACE^−/−^ mice had been backcrossed to C57BL/6J (wildtype) mice that were used as controls. Experiments were conducted on 10- to 12-week-old wildtype and ACE^−/−^ mice.

### Induction of chronic kidney damage with AAI

**Fig. 1:**
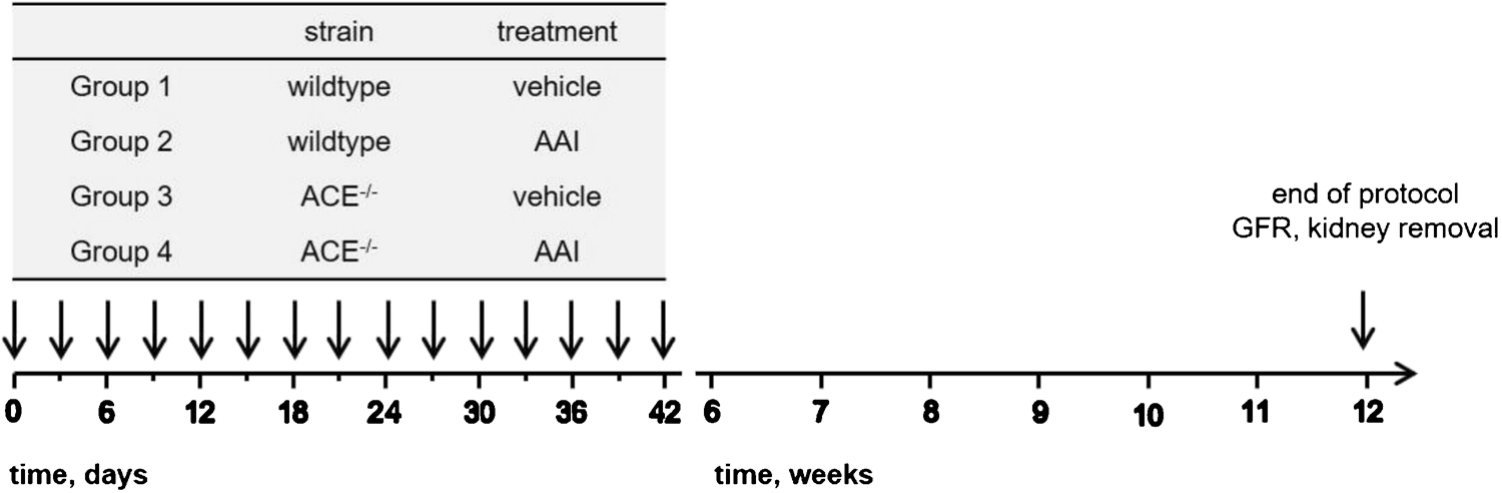
Experimental protocol. AAI, aristolochic acid I; ACE^−/−^, C57BL/6J-*tm*^*(ACE3/3)*^ mice; GFR, glomerular filtration rate. Arrows mark the days mice received vehicle or AAI

Chronic kidney damage was induced using 3 mg AAI (Sigma Aldrich, Munich, Germany) per kg body weight (i.p.) every 3 days for 6 weeks followed by 6 weeks without treatment [29] (Fig. 1). The mice were randomized to four groups. Groups 1 (wildtype mice) and 3 (ACE^−/−^ mice) received vehicle (dimethyl sulfoxide) only, whereas groups 2 (wildtype mice) and 4 (ACE^−/−^ mice) received AAI as described above. At the end of the protocol, GFR was determined and kidneys were harvested for further analyses.

### Glomerular filtration rate

For measurements of GFR using inulin clearance, the mice were anesthetized with a combination of ketamine and xylazine (12.5 mg mL^−1^ and 2.5 mg mL^−1^, respectively, i.p.). During the protocol for GFR measurements, ketamine was supplemented as needed. After a median skin incision and separation of the subcutaneous fatty tissue of the ventral neck region, polyethylene catheters were implanted into the trachea, carotid artery, and jugular vein. After a second small skin incision in the abdominal region, a catheter was implanted into the urinary bladder. The mice were mechanically ventilated (tidal volume: 160 μL, breathing frequency: 160 min^−1^) and continuously infused via the jugular vein catheter (infusion rate: 2.5 µL min^−1^) [5] with an isotonic sodium chloride solution containing 1% bovine serum albumin and 0.5% inulin. Blood pressure and heart rate were monitored via the carotid artery catheter with a PowerLab™ data acquisition system (ADInstruments, Oxford, UK), and urine was collected via the bladder catheter. After a stabilization period of at least 30 min, a 30-min urine collection was performed. At the end of the protocol, 500 µL of blood was collected from the carotid artery catheter for measuring serum inulin concentration. Inulin clearance (*C*_inulin_) was calculated according to the following formula:

$$C_{\mathrm{inulin}}=\frac{\dot V\times{\left[\mathrm{inulin}\right]}_{\mathrm{urine}}}{{\left[\mathrm{inulin}\right]}_{\mathrm{serum}}}\times\mathrm{kidney}\;\mathrm{weight}^{-1}$$

where $\dot{\text{V}}$ = urine flow given in µL min^−1^, ${\text{[inulin]}}_{\text{urine}}$ = urinary inulin concentration given in mmol L urinary inulin^−1^, ${\text{[inulin]}}_{\text{serum}}$ = serum inulin concentration given in mmol L^−1^ and kidney weight was given in g.

### Serum and urinary inulin concentrations

Serum and urinary inulin concentrations were measured photometrically. Samples of 150 µL of undiluted serum were mixed with the same amount of trichloroacetic acid for protein precipitation and centrifuged (8 min, 14,000 *g*, room temperature); 200 µL of the supernatant was mixed with the same amount of resorcinol, and 300 µL hydrochloric acid was added. After 25 min at 80 °C, the reaction was stopped by cooling the samples on ice. The samples were analyzed in triplicate. Optical density was detected at 492 nm using a microplate reader (FLUOstar OPTIMA; BMG Labtech, Ortenberg, Germany). Serum inulin concentration was determined by linear regression based on a standard curve. To determine urinary inulin concentration, the samples were heated (4 min, 60 °C) and diluted (1:10, 1:20, 1:40, or 1:50). Otherwise, the assay procedure was the same as described above for serum inulin concentration.

### Histology, immunohistochemistry, and quantitative image analysis

After completion of the GFR protocol, kidneys were perfused with phosphate-buffered saline (PBS) and removed for further investigation. One half from each horizontally sectioned kidney was fixed at room temperature in a Bouin solution for 24 h. The other half remained unfixed and was used for determination of specific protein species (see below). After fixation, the kidneys were washed three times for 1 h in 70% ethanol. The kidneys were dehydrated and embedded in paraffin according to standard protocols, and 4-µm sections were cut on a microtome (SM 2000 R; Leica Microsystems, Nussloch, Germany), mounted on glass slides (SuperFrost; Menzel, Braunschweig, Germany), and deparaffinized in xylene and descending ethanol series. The sections were stained either with hematoxylin and eosin (HE) or Masson’s trichrome. Light microscopy was performed using an Olympus BX 50 microscope equipped with a digital camera, UC 30 (Olympus Europe GmbH, Hamburg, Germany).

For immunohistochemistry, the sections were subjected to heat-induced epitope retrieval by boiling sections for 5 min in a 10-mM citric acid buffer (pH 6). Endogenous peroxidase was blocked using the BLOXALL blocking solution (Vector Laboratories). The sections were blocked in 2.5% normal horse serum. Primary polyclonal rabbit anti KIM-1 [LSBio (LifeSpan) Cat# LS-B2103-50, RRID:AB_1508933] antibody diluted to 5 µg mL^−1^ was incubated at 4 °C overnight. The sections were extensively washed in 1 × PBS. Bound primary antibodies were visualized using Vectastain Elite ABC-HRP Kit, peroxidase (Vector Laboratories) with Vectastain DAB Substrate Kit, peroxidase (Vector Laboratories). The sections were counterstained with hematoxylin, cleared in xylene, and mounted in Eukitt (Carl Roth, Karlsruhe, Germany).

Sirius red staining was performed according to Puchtler: 5-µm sections were dehydrated as described above. The sections were incubated for 1 h in 0.1% Sirius red dissolved in a saturated picric acid solution. After two washes in 50% acetic acid, an ascending ethanol series, and clearing in xylene, the sections were mounted in Eukitt (Carl Roth, Karlsruhe, Germany).

Whole-slide images of kidney cross-sections were acquired on a Leica SCN-400 slide scanner using the × 40 objective and exported as.scn files. The whole-slide data was imported to QuPath (v.0.3.0). The respective channel of interest (DAB) was isolated using color deconvolution. Thresholding-based pixel classification was used to quantify the DAB positive and DAB negative areas. The percentage of decellularized tubular segments, meaning tubules with denuded basement membranes without cellular coverage, was evaluated in Sirius red-stained sections and quantified for every kidney section in five individual high-power magnification fields of view.

### Renal AGT, renin, ACE, ACE2, and Mas protein

To determine the abundance of specific proteins, renal tissue was homogenized in liquid nitrogen and lysed in a radioimmunoprecipitation assay buffer [containing 0.05 mol L^−1^ Tris–HCl, 0.3 mol L^−1^ NaCl, 0.1% sodium dodecyl sulfate (SDS), 1% NP-40, 0.001 mol L^−1^ phenylmethyl sulfonyl fluoride (PMSF), 1% sodium pyrophosphate decahydrate, and 10 μL mL^−1^ protease inhibitor] or in a 4-(2-hydroxyethyl)-1-piperazineethanesulfonic acid (HEPES) buffer (containing 0.1 mol L^−1^ HEPES, 0.1 mol L^−1^ NaF, 0.1 mol L^−1^ ethylene diamine tetraacetate (EDTA), 0.01 mol L^−1^ sodium metavanadate, 1% triton X-100, 0.002 mol L^−1^ PMSF, 0.1 mol L^−1^ sodium pyrophosphate decahydrate, 10 μL mL^−1^ protease inhibitor). Protein concentration was determined by the bicinchoninic acid assay method (Pierce™ BCA Protein Assay Kit; Thermo Scientific™, Schwerte, Germany). For immunoblotting of AGT, renin, ACE, ACE2, or Mas, equal amounts of protein (40 µg) was denatured in a sample buffer (5 min, 95 °C). Total protein was separated on 4–15% SDS polyacrylamide gel (Criterion TGX Stain-Free Precast Gels; Bio-Rad Laboratories GmbH, Munich, Germany). After separation, proteins were transferred to a nitrocellulose membrane, blocked with 5% bovine serum albumin or Roti Block (Roth, Karlsruhe, Germany), and incubated with antibodies against AGT (1:4000, R and D Systems Cat# AF6966, RRID:AB_10971952, Inc., Minneapolis, USA), renin (1:2000, ABIN301824, antibodies-online, Aachen, Germany), ACE (1:1000, orb216086; Biorbyt Ltd., Cambridge, UK), ACE2 (1:1000, Abnova Cat# PAB13443, RRID:AB_10549736, Taipei City, Taiwan) and Mas (1:2000, Novus Cat# NBP1-78,444, RRID:AB_11039164, Wiesbaden-Nordenstadt, Germany), respectively, followed by a secondary horseradish peroxidase (HRP)-conjugated antibody (goat anti-rabbit HRP Conjugate, Bio-Rad Cat# 170–6515, RRID:AB_11125142, Munich, Germany or rabbit anti-goat HRP Conjugate, Millipore Cat# AP106P, RRID:AB_9241, Darmstadt, Germany).

Immunoreactive bands were detected using an enhanced chemiluminescence kit (ECL Plus; Amersham Pharmacia Biotech, Buckinghamshire, UK) and quantified using an imaging system and software (ChemiDoc™ XRS + and Image Lab; Bio-Rad Laboratories GmbH, Munich, Germany). Abundances of specific protein species were determined relative to total protein abundance normalized to the relative protein abundance of vehicle-treated wildtype mice.

### Renal and serum enzymatic ACE activities

Renal and serum enzymatic ACE activities were determined using a commercially available kit (ACE Color; Fujirebio, Hannover, Germany) according to the manufacturer’s instructions and adapted to 96-well plates. To determine the renal ACE activity, 10–36 mg of renal tissue was suspended in a lysis buffer [containing 50 mmol L HEPES, 0.5% Triton X-100, 0.025 mmol L ZnCl_2_, 150 mmol L^−1^ NaCl, 1 mmol L^−1^ PMSF, 1 tablet EDTA-free protease inhibitor cocktail (cOmplete Mini, Roche, Basel, Switzerland); 10 µL Lysis buffer/1 mg tissue]. Samples were homogenized, incubated for 60 min at 4 °C, and centrifuged (15 min, 8000 *g*, room temperature). The supernatant was removed and assayed undiluted. To determine the serum ACE activity, samples were diluted (1:3).

### Renal Ang(1–7) concentration

Renal Ang(1–7) concentration was determined using a commercially available kit [Mouse Angiotensin 1–7 (Ang1–7) ELISA Kit; Cusabio Technology Llc, Houston, TX, USA] according to the manufacturer’s instructions. To determine renal Ang(1–7) concentration, 30 mg of renal tissue was suspended in 300 µL of PBS and stored overnight at −20 °C. After two freeze–thaw cycles, the homogenates were centrifuged (5 min, 5000 *g*, 4 °C). The supernatant was removed, diluted (1:20 or 1:40), and assayed immediately.

### Statistical analyses

The results are expressed as means ± SEM. The data were analyzed by two-way ANOVA with post-hoc Bonferroni test. A value of *p* < 0.05 was considered statistically significant. Statistical analyses and graphs were performed with Prism 9.2.0 (®1992–2021; GraphPad Software Inc., La Jolla, CA, USA).

## Results

### Renal ACE in ACE^−/−^ mice

**Fig. 2:**
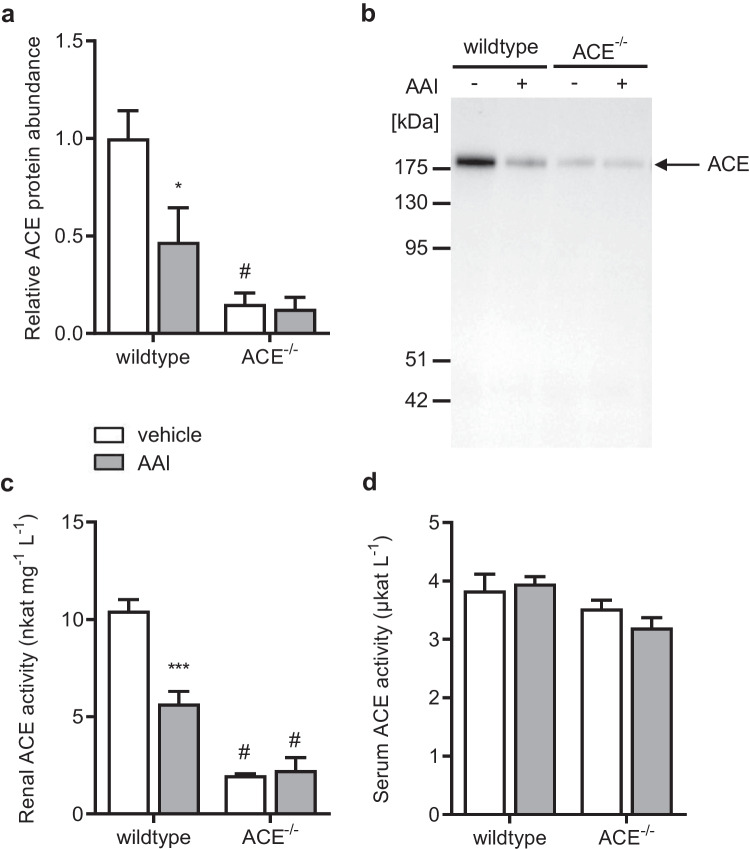
**a** Renal angiotensin I-converting enzyme (ACE) protein abundance normalized to total protein, **b** Western blot from kidney tissue using an antibody against ACE, **c** renal ACE activity, and **d** serum ACE activity in the wildtype and ACE^−/−^ mice treated with vehicle or aristolochic acid I (AAI). ACE^−/−^  = C57BL/6J-*tm*^*(ACE3/3)*^ mice, *n* = 6–8 per group, two-way ANOVA: **a** treatment *p* < 0.05; strain *p* < 0.001; interaction *n.s.*; **c** treatment *p* < 0.01; strain *p* < 0.0001; interaction *p* < 0.01; **d** treatment *n.s.*; strain *p* < 0.05; interaction *n.s.*; Bonferroni test: *p < 0.05, ***p < 0.001 vs. vehicle; #*p* < 0.05 vs. wildtype

As expected, renal ACE protein abundance in the ACE^−/−^ mice was only a fraction of that in the wildtype mice (Fig. 2a, white columns, 2b). Specifically, in the ACE^−/−^ mice, renal ACE protein abundance was down to about 15% of that in the wildtype mice. In accordance with the low renal ACE protein abundance in the ACE^−/−^ mice, renal ACE activity was also reduced to about 10% of that in the wildtype mice (Fig. 2c, white columns). In contrast to the local ACE activity in the renal tissue, serum ACE activity was similar in both strains (Fig. 2d, white columns).

### Effects of AAI on renal morphology and function

**Fig. 3:**
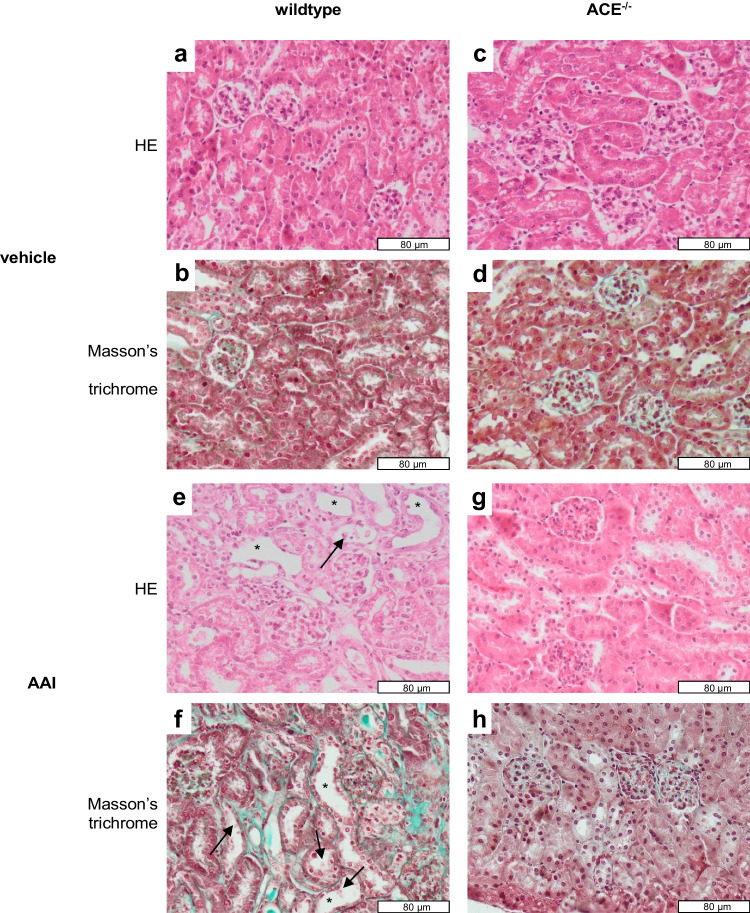
Kidney paraffin sections stained with hematoxylin and eosin (HE) or Masson’s trichrome (magnification × 40). Kidneys were obtained from wildtype or ACE^−/−^ mice that had been treated with vehicle or aristolochic acid I (AAI). ACE^−/−^, C57BL/6J-*tm*^*(ACE3/3)*^ mice. Arrows mark tubular cell loss and asterisks mark luminal dilation

Kidneys from the vehicle-treated wildtype and ACE^−/−^ mice were similar with respect to macroscopic and histological appearance (Fig. 3a–d) as well as renal weight (wildtype 0.39 ± 0.02 g, ACE^−/−^ 0.38 ± 0.02 g, *n.s.*). In contrast, kidneys from AAI-treated wildtype mice appeared paler on macroscopic inspection and showed more severe histological lesions than kidneys from AAI-treated ACE^−/−^ mice. Thus, kidneys from the AAI-treated wildtype mice exhibited profound tubular cell loss and luminal dilation (Fig. 3e and 3f), whereas these structures appeared to be essentially intact except for some minor proliferation of connective tissue in the AAI-treated ACE^−/−^ mice (Fig. 3g and 3h). Specifically, the percentage of decellularized tubular segments, meaning tubules with denuded basement membranes without cellular coverage, was 16.4 ± 1.8% in kidneys from the AAI-treated wildtype mice vs. only 4.7 ± 1.1% in kidneys from the AAI-treated ACE^−/−^ mice (*p* < 0.001).

In general, kidneys from the AAI-treated mice were significantly smaller than kidneys from the vehicle-treated mice (wildtype: 0.39 ± 0.02 g vs. 0.22 ± 0.01 g, ACE^−/−^: 0.38 ± 0.02 g vs. 0.23 ± 0.01 g; all *p* < 0.001), without statistically significant differences between the two strains (wildtype 0.22 ± 0.01 g, ACE^−/−^ 0.23 ± 0.01 g; *n.s.*).

**Fig. 4:**
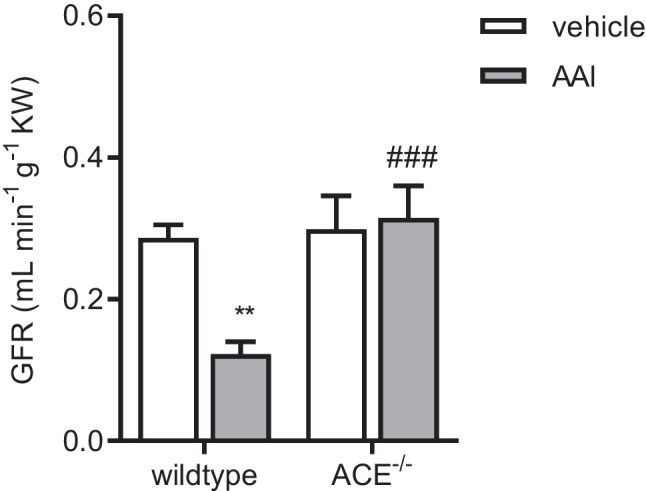
Glomerular filtration rate (GFR) in the wildtype and ACE^−/−^ mice treated with vehicle or aristolochic acid I (AAI). ACE^−/−^, C57BL/6J-*tm*^*(ACE3/3)*^ mice, KW, kidney weight, *n* = 6–8 per group, two-way ANOVA: treatment *p* < 0.05; strain *p* < 0.01; interaction *p* < 0.05; Bonferroni test: **p < 0 .01 vs. vehicle; ###*p* < 0.001 vs. wildtype

Glomerular filtration rate (GFR), as measured by inulin clearance, was similar in the vehicle-treated wildtype and ACE^−/−^ mice (Fig. 4). AAI significantly decreased GFR by about 55% in the wildtype but not in the ACE^−/−^ mice, where it remained essentially unaltered.

**Fig. 5:**
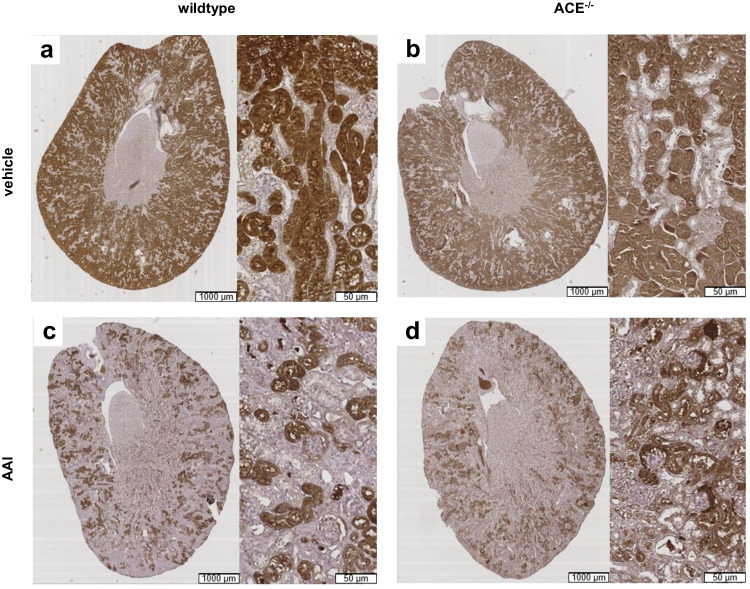
Kidney paraffin sections incubated with primary kidney injury molecule (KIM)-1 antibody. Kidneys were obtained from the wildtype or ACE^−/−^ mice that had been treated with vehicle or aristolochic acid I (AAI). ACE^−/−^, C57BL/6J-*tm*^*(ACE3/3)*^ mice

In our model of chronic renal failure, the expression of the acute injury marker, kidney injury molecule (KIM)-1, was similar in kidneys from the vehicle-treated wildtype and ACE^−/−^ mice (Fig. 5a and 5b). AAI led to a statistically significant reduction of KIM-1 expression in the wildtype but not in the ACE^−/−^ mice (Fig. 5c and 5d; wildtype: 63.18 ± 1.08% vs. 33.68 ± 2.66% KIM-1-positive area, *p* < 0.05; ACE^−/−^: 62.55 ± 1.25% vs. 34.45 ± 2.48% KIM-1-positive area, *n.s.*).

### Effects of AAI on renal angiotensinogen, renin, and ACE

**Fig. 6:**
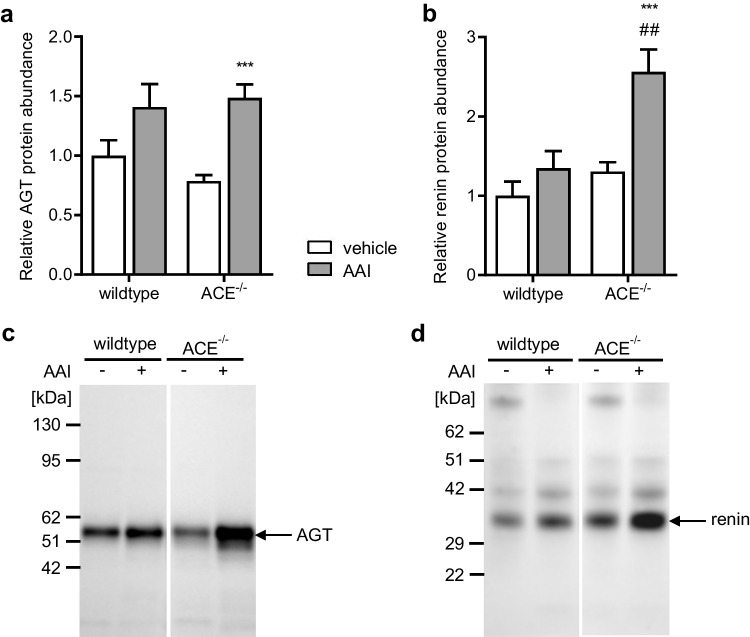
**a** Renal angiotensinogen (AGT) protein abundance, **b** renal renin protein abundance normalized to total protein, and **c** Western blot from kidney tissue using an antibody against AGT and **d** against renin in the wildtype and ACE^−/−^ mice treated with vehicle or aristolochic acid I (AAI). ACE^−/−^, C57BL/6J-*tm*^*(ACE3/3)*^ mice, *n* = 6–10 per group, two-way ANOVA: **a** treatment *p* < 0.001; strain *n.s.*; interaction *n.s.*; **b** treatment *p* < 0.001; strain *p* < 0.001; interaction *p* < 0.05; Bonferroni test: ***p < 0.0001 vs. vehicle; ##*p* < 0.01 vs. wildtype

Renal angiotensinogen (AGT) protein abundance was similar in kidneys from the vehicle-treated wildtype and ACE^−/−^ mice (Fig. 6a and 6c). AAI significantly increased renal AGT protein abundance in the ACE^−/−^ mice. There was also a slight increase in renal AGT protein abundance in the AAI-treated wildtype mice, but this effect did not reach statistical significance.

Renal renin protein abundance was similar in the vehicle-treated wildtype and ACE^−/−^ mice (Fig. 6b and 6d). AAI significantly increased renal renin protein abundance in the ACE^−/−^ mice but not in the wildtype mice.

As intended and as mentioned above, the ACE^−/−^ mice showed only minor renal ACE protein abundance compared to the wildtype mice (Fig. 2a and 2b). AAI strongly and significantly decreased renal ACE protein abundance in the wildtype mice, whereas it did not significantly affect the already very low renal ACE protein abundance in the ACE^−/−^ mice. In accordance with these results, AAI also significantly decreased renal ACE activity in the wildtype mice but did not significantly affect renal ACE activity in the ACE^−/−^ mice (Fig. 2c). Furthermore, AAI did not significantly affect serum ACE activity in either strain (Fig. 2d).

### Effects of AAI on the renal ACE2/Ang(1–7)/Mas axis

**Fig. 7:**
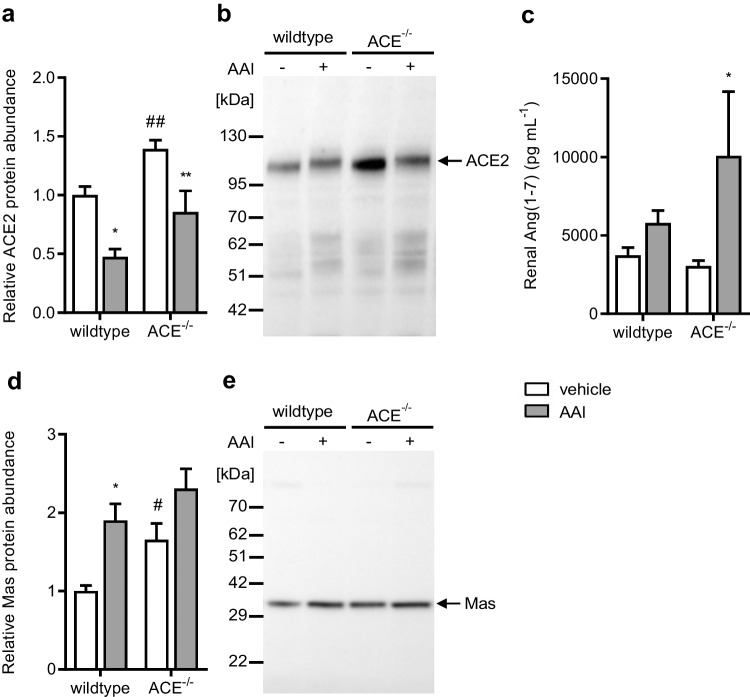
**a** Renal angiotensin I-converting enzyme 2 (ACE2) protein abundance normalized to total protein, **b** Western blot from kidney tissue using an antibody against ACE2, **c** renal angiotensin (Ang) (1–7) concentration, **d** renal Mas protein abundance normalized to total protein, and **e** Western blot from kidney tissue using an antibody against Mas in the wildtype and ACE^−/−^ mice treated with vehicle or aristolochic acid I (AAI). ACE^−/−^, C57BL/6 J-*tm*^*(ACE3/3)*^ mice, *n* = 6–10 per group, two-way ANOVA: **a** treatment *p* < 0.0001; strain *p* < 0.001; interaction *n.s.*; **c** treatment *p* < 0.05; strain *n.s.*; interaction *n.s.*; **d** treatment *p* < 0.01; strain *p* < 0.05; interaction *n.s.*; Bonferroni test: *p < 0.05, **p < 0.01 vs. vehicle; #*p* < 0.05, ##*p* < 0.01 vs. wildtype

Renal ACE2 protein abundance was significantly higher in the vehicletreated ACE^−/−^ mice than in the vehicle-treated wildtype mice (Fig. 7a and 7b). AAI similarly and significantly decreased renal ACE2 protein abundance in both strains.

Renal Ang(1–7) concentration was similar in the vehicle-treated wildtype and ACE^−/−^ mice (Fig. 7c). AAI significantly increased renal Ang(1–7) concentration in the ACE^−/−^ mice but not in the wildtype mice.

Renal Mas protein abundance was significantly higher in the vehicle-treated ACE^−/−^ mice than in the vehicle-treated wildtype mice (Fig. 7d and 7e). AAI increased renal Mas protein abundance in the wildtype mice, roughly to that in the vehicle-treated ACE^−/−^ mice. AAI also slightly increased renal Mas protein abundance in the ACE^−/−^ mice, but this effect did not reach statistical significance.

## Discussion

The present study shows that renal ACE deficiency protects against AAN, a new type of nephropathy that has recently evoked major research efforts due to its widespread occurrence and its often malignant course [11, 15, 17, 18, 20, 33, 65, 66, 72, 73]. It is currently widely accepted that the renal RAS plays a major role in the pathophysiology of many types of kidney disease and of systemic arterial hypertension [19, 35, 40, 41, 43, 45, 50, 54, 55]. Thus, not only are ACE inhibitors indispensable drugs for the clinical treatment of kidney diseases [71], but the selective experimental removal of components of the renal RAS, including renal ACE and renal AT_1_R, was also shown to protect mice against experimentally induced arterial hypertension [26, 25, 13,1 2]. Whether renal ACE deficiency also protects against nephropathy has not yet been investigated.

For our study, we used a strain of renal ACE-deficient mice, kindly provided by Dr. Romer Gonzalez-Villalobos, Dept. of Biomedical Sciences, Cedars-Sinai Medical Center, Los Angeles, CA, USA. In keeping with previous reports[9, 26], these mice had essentially normal renal histology and function, while renal ACE abundance was reduced to only about 15% of that in the wildtype controls. Furthermore, reflecting the low renal ACE abundance, renal ACE activity was also dramatically reduced in the ACE^−/−^ mice to as low as about 10 percent of that in the wildtype mice. Nevertheless, due to liver-derived ACE as ACE gene transcription was set under the control of the albumin promoter, serum ACE activity in the ACE^−/−^ mice was normal, confirming previous reports from the literature [9, 10, 26].

When challenged with AAI at 3 mg kg^−1^, i.p., every third day for 6 weeks, the wildtype mice developed severe nephropathy with profound tubular cell loss and luminal dilation as well as significantly reduced GFR. The dose regimen for AAI applied in the present study was deployed previously by another group [29] obtaining similar results with respect to the development of nephropathy. Apparently, the nephrotoxic effects of AAI on mice strongly depend on dosage and application regime and may range from minor alterations to end-stage renal insufficiency [29]. Whereas many studies investigating the effects of AAI on renal function in experimental animals relied on plasma creatinine concentration or proteinuria alone or in combination as a method to assess renal function [14, 67, 68], we chose to apply the more robust and meaningful technique of measuring inulin clearances.

In contrast to the severe effects of AAI on renal morphology and function seen in the wildtype mice, there were only limited signs of histological renal lesions, including minor proliferation of connective tissue, and there was no statistically significant reduction in GFR in the ACE^−/−^ mice. Our immunohistochemical data on the kidney injury marker KIM-1 may appear counterintuitive on first sight as AAI-induced renal damage was associated with a decrease rather than an increase in KIM-1 expression in both strains. It should be noted, however, that KIM-1 is a fast reacting marker of acute rather than chronic renal damage, which can only be detected in renal tubular cells as long as they remain vital [31]. Thus, the decreased KIM-1 expression in our model of chronic renal failure may be explained by the AAI-induced loss of vital tubular cells. In keeping with our histological and functional results, the AAI-induced reduction of KIM-1 expression was statistically significant in the wildtype but not in the ACE^−/−^ mice, albeit the absolute differences between both strains were small.

Thus, it appears that renal ACE deficiency may convey protective effects against aristolochic acid-induced nephropathy, triggering the question as to the underlying molecular mechanisms. In this respect, specific interventions with several molecular signaling pathways outside the renal RAS have been identified that may convey reno-protective effects on AAN, including inhibition of p53 [73], blockade of the TGF-β-mediated signaling pathway [48, 72], and increase in NO bioavailability [18, 33].

On the other hand, in an experimental study on rats by Debelle et al. [16], pharmacological RAS blockade with the combination of an ACE inhibitor and an AT_1_R antagonist failed to improve renal function in AAI-induced nephropathy. Whereas this result appears to be in contrast to our present findings, there are several differences concerning experimental design and species between the two studies that may explain the discrepancy. Thus, in the study by Debelle et al. [16], the RAS blockers were administrated in drinking water during the course of the study, whereas in our study, there was genetically induced and therefore lifelong renal ACE deficiency. Furthermore, Debelle et al. [16] applied AAI at a single dose of 10 mg kg^−1^ body weight, a dose regimen that in our hands resulted in acute and rapidly progressive nephropathy (data not shown) with high mortality in contrast to the more chronic type of kidney disease as elicited with repeated applications of AAI in smaller doses (3 mg kg^−1^ body weight) in our study. Finally, species differences between rats and mice, with rats being more susceptible to kidney disease than mice [24, 32, 36, 42, 44, 49], may have contributed to the divergent results between the two studies.

It is currently well accepted that in many tissues, including the kidney, the classical ACE/AngII/AT_1_R axis is complemented by the alternative ACE2/Ang(1–7)/Mas axis [3, 56–58]. In contrast to the former, which tends to worsen ensuing renal damage, the latter axis may entail predominantly protective effects on the kidney [1, 22, 52, 61]. We therefore hypothesized that in the face of renal ACE deficiency more of locally generated AngI may be converted by renal ACE2 to Ang(1–7), thus shifting the balance away from the “detrimental” ACE/AngII/AT_1_R axis to the “beneficial” ACE2/Ang(1–7)/Mas axis. Our finding that renal ACE2 and Mas abundances were significantly increased under baseline conditions in the ACE^−/−^ compared to the wildtype mice supports this hypothesis, although renal Ang(1–7) concentration was initially not significantly different between the two strains.

Further hints toward a possible shift of balance between the two RAS axes away from the renal disease-promoting toward the reno-protective pathway in the ACE^−/−^ mice were obtained after the induction of AAI-induced nephropathy. Under these conditions, the beginning of the local RAS cascade was activated in the ACE^−/−^ but not in the wildtype mice as indicated by significantly increased renal AGT and renin abundances in the former strain. As renin cleaves AngI from AGT, and the ACE^−/−^ mice were, by design, deficient of renal ACE but not of renal ACE2, which was in fact elevated in the ACE^−/−^ compared to the wildtype mice, these effects ultimately resulted in a significantly increased renal Ang(1–7) concentration in the ACE^−/−^ compared to the wildtype mice. Since baseline renal Mas abundance was significantly higher in the ACE^−/−^ than in the wildtype mice, the increased renal Ang(1–7) concentration after AAI coincides with increased renal levels of its specific receptor. While these findings support the hypothesis that the protection of renal ACE-deficient mice from AAI-induced nephropathy may have been partly due to the activation of the local ACE2/Ang(1–7)/Mas axis in the kidney, parts of our data are not equally suggestive in this respect. Thus, in agreement with a previous report also using C57BL/6J mice [63], renal ACE2 protein abundance was downregulated in response to AAI in both strains. Furthermore, renal Mas protein abundance was similar in the AAI-treated wildtype and ACE^−/−^ mice. Further studies, including those involving pharmacological Mas receptor antagonists, may help to clarify these issues.

The molecular mechanisms by which activation of the ACE2/Ang(1–7)/Mas pathway may protect the kidney from AAI-induced nephropathy are currently not completely understood. In this respect, Mas activation has been reported to inhibit signal transduction pathways that are known to promote AAI-induced detrimental effects on the kidney, such as apoptosis and fibrosis [2, 46, 53, 59, 69]. These pathways may include MAP kinases [53] such as ERK1/2, p38, and JNK, as well as the TGF-β/NFκB axis [69] and other mechanisms [2, 46, 59]. The inhibition of some of these pathways has been shown to provide reno-protective effects on AAI-induced nephropathy [46, 48].

While with AGT and renin the renal abundances of two major components of the local RAS increased in AAI-induced nephropathy, matching similar experimental [23, 27, 51, 62] and clinical observations [4, 23, 64] in other types of chronic nephropathy, renal ACE abundance, and activity in wildtype mice decreased to about half of their baseline values. Given the fact that PTECs are a major source of local ACE in the kidney [7], the latter effect agrees well with our histological findings showing substantial tubular cell loss after AAI in the wildtype mice.

The notion that the AAI-induced decrease in renal ACE abundance and activity is due to tubular cell loss is supported by the lack of a similar effect in the ACE^−/−^ mice, which showed only minor histological lesions and essentially normal tubular structures, although it needs to be taken into account that in the ACE^−/−^ mice renal ACE abundance and activity were already very low to begin with.

In contrast to local ACE activity in the kidney, which was significantly reduced after AAI, serum ACE activity was not significantly affected by the nephrotoxic agent. In this respect, several studies [6, 29] showed that AAI predominantly affects the kidney and has minor toxic effects on hepatic functions, including the synthesis and release of ACE in the ACE^−/−^ mice.

Taken together, our data show that renal ACE deficiency protects against AAI-induced nephropathy. While the molecular mechanisms underlying this effect are currently not completely understood, our data suggest that in the face of renal ACE deficiency, AAI may activate the ACE2/Ang(1–7)/Mas pathway, which in turn may deploy its well-known reno-protective effects.

## Data Availability

The data that support the findings of this study are available from the corresponding author upon reasonable request.
